# MAPK uncouples cell cycle progression from cell spreading and cytoskeletal organization in cycling cells

**DOI:** 10.1007/s00018-012-1130-2

**Published:** 2012-08-25

**Authors:** Coert Margadant, Lobke Cremers, Arnoud Sonnenberg, Johannes Boonstra

**Affiliations:** 1 Department of Cell Biology, Faculty of Sciences, University of Utrecht, Padualaan 8, 3584 CH Utrecht, The Netherlands; 2 Department of Cell Biology, The Netherlands Cancer Institute, Plesmanlaan 121, 1066 CX Amsterdam, The Netherlands

**Keywords:** Actin cytoskeleton, Cell cycle progression, Cell spreading, Cyclin, Focal adhesion, G1-phase, Integrin, Mitogen-activated protein kinase, Mitosis

## Abstract

Integrin-mediated cytoskeletal tension supports growth-factor-induced proliferation, and disruption of the actin cytoskeleton in growth factor-stimulated cells prevents the re-expression of cyclin D and cell cycle re-entry from quiescence. In contrast to cells that enter the cell cycle from G0, cycling cells continuously express cyclin D, and are subject to major cell shape changes during the cell cycle. Here, we investigated the cell cycle requirements for cytoskeletal tension and cell spreading in cycling mammalian cells that enter G1-phase from mitosis. Disruption of the actin cytoskeleton at progressive time-points in G1-phase induced cell rounding, FA disassembly, and attenuated both integrin signaling and growth factor-induced p44/p42 mitogen-activated protein kinase activation. Although cyclin D expression was reduced, the expression of cyclin A and entry into S-phase were not affected. Moreover, expression of cyclin B1, progression through G2- and M-phase, and commitment to a new cell cycle occurred normally. In contrast, cell cycle progression was strongly prevented by inhibition of MAPK activity in G1-phase, whereas cell spreading, cytoskeletal organization, and integrin signaling were not impaired. MAPK inhibition also prevented cytoskeleton-independent cell cycle progression. Thus, these results uncouple the requirements for cell spreading and cytoskeletal organization from MAPK signaling, and show that cycling mammalian cells can proliferate independently of actin stress fibers, focal adhesions, or cell spreading, as long as a threshold level of MAPK activity is sustained.

**Electronic supplementary material:**

The online version of this article (doi:10.1007/s00018-012-1130-2) contains supplementary material, which is available to authorized users.

## Introduction

Cell cycle progression in normal mammalian cells requires both growth factor stimulation and cell adhesion to the extracellular matrix (ECM) mediated by transmembrane heterodimeric αβ receptors called integrins [1, 2]. Engagement of virtually all integrins leads to the assembly of large macromolecular complexes called focal adhesions (FAs), at least in cells in culture, where they connect to actin stress fibers. In this way, integrins generate intracellular tension, mediate cell spreading over the substrate, and initiate several signaling pathways [3, 4]. Disruption of stress fibers with pharmacological agents prevents integrin-mediated cell spreading, and inhibits growth-factor-induced re-entry into G1-phase from quiescence [5–14]. Cell cycle arrest upon cytoskeletal disruption is characterized by a failure to downregulate the cell cycle inhibitor p27^KIP1^, to induce sustained p44/p42 mitogen-activated protein kinase (MAPK) phosphorylation and activation, and to induce expression of D-type cyclins, which together with cdk4/6 are essential factors for promoting G1/S-phase progression [12–15]. The same is observed upon loss of adhesion, or restriction of cell spreading on micro-patterned surfaces [16–18].

Induction of cyclin D expression is crucial for growth factor-stimulated re-entry into G1-phase from quiescence, and is the main step that is sensitive to cytoskeletal tension [5, 6, 19–22]. Overexpression of cyclin D1 can rescue proliferation in non-adherent cells, and is often associated with the anchorage-independent growth of tumor cells [23–26]. It thus seems that the requirement of cell spreading for proliferation is not absolute, and that cell cycle progression and cell spreading can be disconnected as long as cyclin D1 is expressed. Indeed, normal G0- to S-phase progression in fibroblasts depends on integrin-sustained Rho activation, stress fiber formation and cell spreading, which leads to mid-G1-phase induction of cyclin D expression [27, 28]. However, upon inhibition of Rho signaling, a Rac/Cdc42-driven pathway induces early expression of cyclin D and shortening of G1 phase, and allows for cell cycle progression in the absence of stress fibers or cell spreading [28]. Hence, depending on the circumstances, cells may switch between a tension-dependent and -independent mode of proliferation, provided that there is a signal to induce cyclin D.

Consistent with such a model, we have previously shown that in cycling cells (i.e., as opposed to cells entering G1-phase from quiescence), which express cyclin D and contain low levels of cell cycle inhibitors, prevention of post-mitotic stress fiber assembly and concomitant cell spreading does not inhibit progression through G1-phase [29]. We hypothesize that after cytokinesis, cells can commit to a new cycle either in a high-tensional state with extensive stress fibers and FAs, or rounded without extensive stress fibers and FAs, depending on the environmental requirements and/or restraints. Possibly, cell spreading elicits an irreversible cell-cycle program so that artificial cell rounding will inhibit cell cycle progression, which would explain why quiescent, spread cells do not enter the cell cycle upon cytoskeletal disruption. In addition, M-to-S-phase progression may only require cytoskeletal integrity and cell spreading during a discrete time-window, as has been observed for capillary endothelial cells that re-enter G1-phase from quiescence [30].

In the present study, we explored the requirements for cell spreading, stress fibers, FAs, and MAPK signaling in cell cycle progression in cycling cells, as well as the cross-talk between MAPK, cell spreading and the cytoskeleton. We have focused on cell cycle progression supported by fibronectin (FN), as most studies concerning integrin-mediated cytoskeletal tension and proliferation center on FN-binding integrins, most importantly integrin α5β1 [27, 28, 31, 32]. To obtain synchronized cycling cells, we have collected cells from asynchronously growing cultures of N2A and CHO cells by mitotic shake-off, which yields high numbers of cells in cytokinesis without the use of metaphase-blocking agents such as nocodazole, and we have previously extensively characterized cell cycle regulation in these cells [29, 33, 34]. Although both cell lines are mitogen- and adhesion-dependent, we also investigated cell cycle progression in non-transformed mouse GEβ1 cells, as the tensional requirements for cell cycle progression are frequently lost in transformed cells [5].

## Materials and methods

### Antibodies and other materials

CCD, DMSO, FN, UO126, and TRITC-conjugated phalloidin were from Sigma (Steinheim, Germany). BrdU was from Invitrogen, DAPI was from Boehringer-Mannheim, and LB was from Calbiochem (San Diego, CA). CCD and LB were prepared as 1 mg/ml stock solutions in DMSO, and further diluted in culture medium to the indicated concentrations. Antibodies used in this study were directed against BrdU (Bu20a from DAKO, Carpinteria, CA), Cyclin A (Oncogene), Cyclin B1 (Santa Cruz), Cyclin D1/D2 (Upstate Biology), FAK (Transduction Laboratories), p42 MAPK (Upstate Biology), paxillin (Transduction Laboratories), P(p44/p42) MAPK (Cell Signalling Technology), P(Y) (4G10; a kind gift of Dr. K. Wilhelmsen), P(Y397)FAK (Invitrogen), P(Y925)FAK (Biosource), P(Y31)paxillin and P(Y118)paxillin (Biosource), securin (Abcam), and vinculin (Sigma). FITC-, TRITC-, CY5-, and Hrp-conjugated secondary antibodies were purchased from Jackson ImmunoResearch Laboratories.

### Cell culture and synchronization

N2A (Neuro2A) cells were derived from a spontaneous murine neuroblastoma, CHO cells were isolated from the ovary of an adult Chinese hamster, and GEβ1 cells were generated by stable expression of the integrin β1-subunit into epithelial GE11 cells derived from the β1-null mouse embryo [35]. All cell lines were maintained in DMEM supplemented with 10% FCS (Gibco) and penicillin/streptomycin (100 U/ml) at 37°C and 5 % CO_2_. Mitotic cells were obtained by shaking asynchronously growing cell cultures firmly by hand for 1 min at 37°C, and collecting the medium as described previously [36]. After shake-off, mitotic cells were released on coverslips (Menzel, Germany) or in tissue-culture plates (Corning, NY) at a density of 1.5 × 10^4^ cells/cm^2^. Coverslips and tissue-culture plates were coated with FCS and FN (10 μg/ml) over night at 37°C.

### Western blotting

Cells were washed once with ice-cold PBS and subsequently lysed on ice in RIPA buffer (20 mM Tris/HCl (pH 7.5), 150 mM NaCl, 1 % Triton X-100, 1 % sodium desoxycholate, 0.1 % SDS, 1 mM EDTA, 100 mM NaF, 1 mM benzamine, 1 mM PMSF, and 1 mM Na3VO4). Collected lysates were cleared for 2 min at 8,000 × *g* and protein amounts were measured with a Bradford assay using a Bio-Rad Novapath microplate reader. Equal amounts of protein were fractionated on 10 or 15 % gels, after which they were electrophoretically transferred to PVDF membranes (Boehringer-Mannheim, Indianapolis, IN) according to standard procedures. The membranes were blocked with 2 % BSA in PBS containing 0.1 % Tween-20 (Sigma), prior to incubation for 1 h with primary antibodies. After washing, the membranes were incubated for 1 h with secondary antibodies, washed again, and immunoreactivity was detected using ECL reagents (Perkin-Elmer).

### Microscopy

Cell morphology in tissue-culture plates was visualized using a Zeiss microscope (Axiovert 25) at 10× (NA 0.25) and 20× (NA 0.3) magnification. Images were captured on a CCD camera (Axiocam MRC), using Mr. Grab 1.0 software (Zeiss). Time-lapse video microscopy was performed on a Widefield CCD system using a 10× dry lens objective (Carl Zeiss MicroImaging, and images were captured every 15 min at 37°C and 5 % CO_2_. For immunofluorescence, cells on coverslips were fixed in 4 % paraformaldehyde (PFA) in PBS for at least 15 min, after which they were permeabilized with 0.5 % Triton X-100. For BrdU stainings, the nuclei were subsequently denatured with 2 N HCl for 30 min at 37°C. After washing twice with PBS, cells were blocked in 50 mM glycin in PBS for at least 10 min, and washed twice with 0.2 % gelatin in PBS (PBG). Subsequently, they were incubated with primary antibodies for 1 h, washed three times with PBG, and incubated with secondary antibodies for 1 h. Cells were then washed again three times with PBG, and once with PBS containing DAPI. Coverslips were mounted in Mowiol supplemented with DABCO (Calbiochem), and analyzed on a confocal microscope using 20× (NA 0.7) dry, 40× (NA 1.25), and 63× (NA 1.32) oil objectives (Leica). Images were acquired with AxioVision software (Carl Zeiss MicroImaging), and processed using ImageJ and Adobe Photoshop software. Combined DAPI-phase/contrast images were captured on a Zeiss AxioObserver Z1 inverted microscope equipped with a cooled CCD-camera (Hamamatsu ORCA AG) using AxioVision software.

### BrdU/EdU labeling and quantification

Synchronized cells were released in fresh medium containing BrdU (10 μM) in 96-well plates at a density of 1 × 10^4^ cells per well, and treated with the inhibitors at the indicated time-points. The cells were fixed 14 h after mitosis, and BrdU incorporation was determined using the Cell Proliferation, Enzyme-linked Immunosorbent Assay (ELISA) kit (Boehringer-Mannheim), according to the manufacturer’s instructions. Absorbance was measured on a Bio-Rad Novapath microplate reader 5 min after substrate addition. In each experiment, cells supplemented with BrdU were fixed before S-phase (5 h after mitosis) as a negative control. Independent experiments were performed with six samples for each condition, and each experiment was repeated at least three times. Incorporation of BrdU was also analyzed by immunofluorescence, by incubating cells on coverslips with 10 μM BrdU for the indicated time-points. Cells were then fixed, and incorporated BrdU was detected as described above. Alternatively, cells were incubated on coverslips with 10 μM EdU, and EdU incorporation was visualized using the Click-iT EdU imaging kit (Invitrogen) followed by confocal microscopy, according to the manufacturers’ instructions.

### Analysis of cell spreading, bi-nucleation, and nuclear cyclin D

To determine the cell surface area, synchronized cells were seeded in tissue-culture plates, treated as indicated, and fixed with 4 % PFA at appropriate time-points. Phase/contrast images were acquired as described, and the cell area of ~100 cells per condition was determined using ImageJ software. To determine the bi-nucleation index, synchronized cells were seeded on coverslips or tissue-culture plates and treated as indicated, after which they were washed and released in fresh medium for 1 h, fixed, and labeled with DAPI (5 μg/ml). Phase/contrast and DAPI images were acquired from multiple fields, and in each independent experiment, bi-nucleation was determined for 300 cells. To quantify nuclear cyclin D, synchronized cells were plated on coverslips and treated for the indicated time-points, after which cyclin D1/D2 and nuclei were stained for confocal microscopy as described above. Images were acquired from multiple fields using the same settings, and cyclin D(+)-nuclei were quantified with ImageJ from ~250 cells per experiment.

## Results

### Disruption of the actin cytoskeleton in G1-phase reverses post-mitotic cell spreading and focal adhesion assembly

**Fig. 1:**
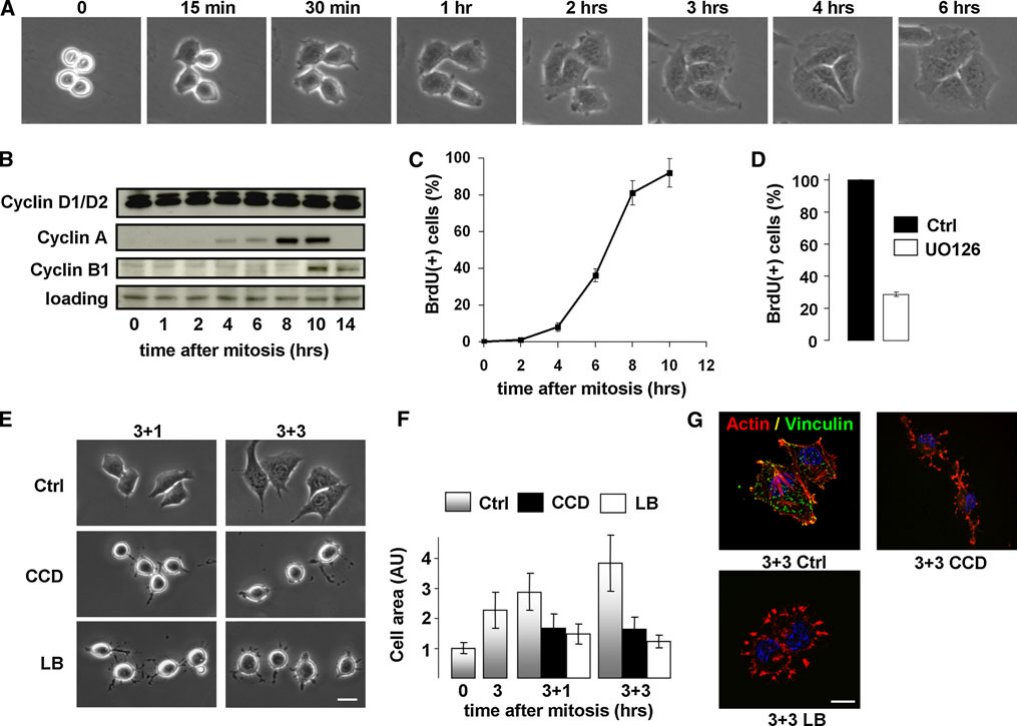
Disruption of actin stress fibers in G1-phase causes cell rounding and FA disassembly. **a** Stills from a time-lapse movie showing N2A cells undergoing cytokinesis and post-mitotic cell spreading. **b** N2A cells were synchronized by mitotic shake-off and released in fresh medium. Cells were lysed at the indicated time-points and the sequential expression of cyclin D1/D2, cyclin A, and cyclin B1 was determined by Western blotting. **c** N2A cells were synchronized by mitotic shake-off and released in the presence of 10 μM BrdU. Progression into S-phase was determined at the indicated time-points by analyzing BrdU incorporation by immunofluorescence. **d** Progression from mitosis to S-phase is largely prevented by inhibition of the MAPK pathway with the inhibitor UO126 (20 μM). **e** N2A cells were synchronized by mitotic shake-off and released in fresh medium. After 3 h, cells were either left untreated (*upper panel*), treated with 500 ng/ml CCD (*mid panel*) or 100 ng/ml LB (*lower panel*), and photographed at 1 or 3 h thereafter. *Bar* 10 μm. **f** Cell area was determined at the indicated time-points from phase-contrast images using ImageJ, and expressed relative to the cell area of mitotic cells. *AU* arbitrary units. **g** N2A cells were synchronized by mitotic shake-off and treated with 500 ng/ml CCD or 100 ng/ml LB 3 h thereafter. Cells were fixed after 3 h of incubation and the nuclei (*blue*), F-actin (*red*), and vinculin (*green*) were visualized with confocal microscopy. *Bar* 10 μm

Here we employ the mitotic shake-off method to collect cells in cytokinesis from asynchronously growing cell cultures. Released cells reattach to the substratum within 15 min and most of them complete cytokinesis within 1 h after synchronization, whereafter cell spreading increases progressively throughout G1-phase (Fig. 1a). Whereas D-type cyclins are expressed in mitotic cells and throughout the entire G1-phase, expression of S-phase promoting cyclin A is induced about 5 h after mitosis, and the G2/M-phase cyclin B1 becomes detectable from 10 h after mitosis (Fig. 1b). The population undergoes the next cytokinesis approximately 16 h after synchronization (data not shown). The induction of cyclin A expression marks the onset of S-phase, and progression to S-phase requires the sustained activity and nuclear translocation of p44/p42 MAPK, as these events are blocked using UO126, an inhibitor of the MAPK pathway (Fig. 1c, d) [33].

To investigate whether cytoskeletal organization and cell spreading are required for cell cycle progression in G1-phase in cycling N2A cells, we used the actin-destabilizing drugs cytochalasin D (CCD) and latrunculin B (LB). Both drugs favor depolymerization of actin filaments but they act by distinct mechanisms; whereas CCD caps the growing ends of actin polymers, LB sequesters actin monomers. Mitotic cells were allowed to re-adhere and spread on FN-coated dishes in fresh medium with serum, after which they were treated with 500 ng/ml CCD or 100 ng/ml LB at progressive time-points in G1-phase. Post-mitotic cell spreading was quantified by measuring the apparent surface area from phase-contrast images. Cell spreading of untreated cells increased from mitosis into G1, and treatment of spread cells in G1-phase (from 3 h after mitosis) with either CCD or LB for 1 h (M + 3_1) or 3 h (M + 3_3) induced cell rounding (Fig. 1e, f). We next analyzed cytoskeletal organization and FA assembly in G1-phase, using the filamentous actin (F-actin)-binding compound phalloidin and an antibody against the FA marker vinculin, which is involved in linking integrins to the actin cytoskeleton. Actin stress fibers associated with FAs were observed in spread cells in G1-phase, and treatment with either drug induced FA loss and dissolution of actin stress fibers, whereas some F-actin was retained in membrane structures left behind after cell rounding (Fig. 1g). Thus, treatment with CCD or LB in G1-phase cells disrupts the normal organization of the actin cytoskeleton, and induces cell rounding and FA disassembly.

### Disruption of the actin cytoskeleton in G1-phase inhibits integrin signaling and growth factor signaling

**Fig. 2:**
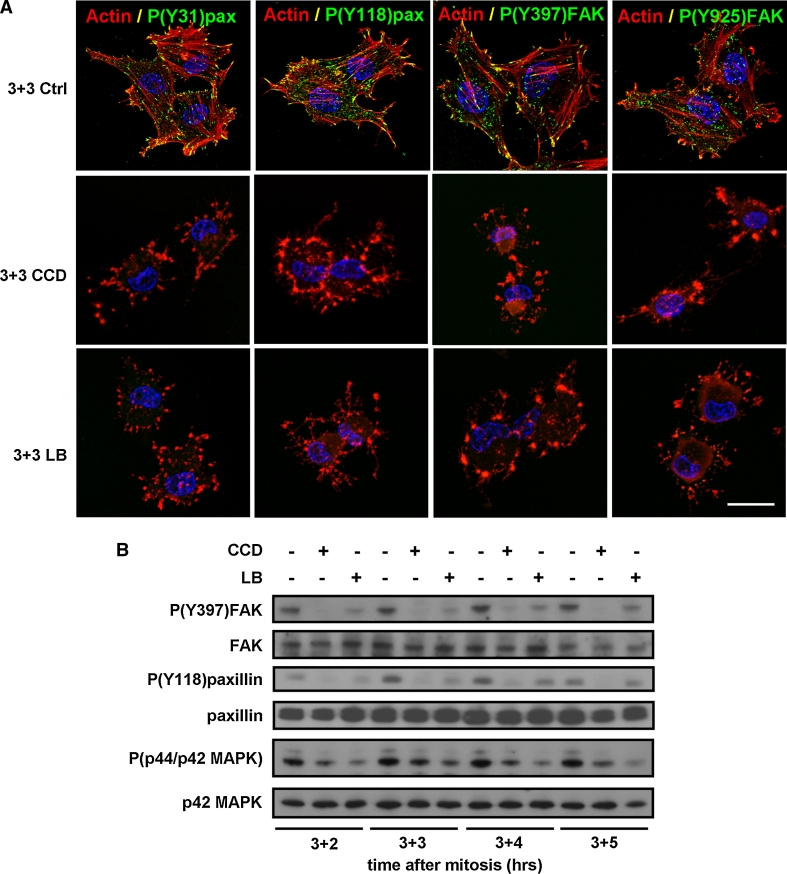
Disruption of actin stress fibers in G1-phase inhibits integrin signaling and growth factor signaling. **a** N2A cells were synchronized by mitotic shake-off, released in fresh medium, and 3 h after synchronization they were either left untreated (*top row*), treated with 500 ng/ml CCD (*middle row*), or treated with 100 ng/ml LB (*bottom row*). Cells were fixed 3 h later and the nuclei (*blue*), F-actin (*red*), and phosphorylation of FAK and paxillin at the indicated residues (*green*) were visualized with confocal microscopy. *Bar* 10 μm. **b** N2A cells were synchronized by mitotic shake-off and 3 h later treated as described. Cells were lysed 2, 3, 4, and 5 h thereafter and autophosphorylation of (Y397)FAK, phosphorylation of (Y118)paxillin, and phosphorylation of p44/p42 MAPK, as well as total levels of FAK, paxillin, and p42 MAPK were investigated by Western blotting

Integrin-mediated re-adhesion and cell spreading after mitosis triggers the auto-phosphorylation of one of the major kinases in FAs, focal adhesion kinase (FAK), on Y397 [29, 37]. Autophosphorylation of FAK triggers the recruitment of Src, which subsequently phosphorylates FAK on Y925. In turn, FAK interacts with the signal transduction adapter protein paxillin, and induces its phosphorylation at Y118 and Y31. To determine whether cytoskeletal disruption and cell rounding induced by CCD or LB in G1-phase cells affects integrin signaling, we treated G1-phase cells 3 h after mitosis with the actin inhibitors, and investigated the phosphorylation of paxillin and FAK by immunofluorescence and Western blotting. Whereas paxillin phosphorylation on Y118 and Y31 and FAK phosphorylation on Y397 and Y925 were clearly visible in FAs in untreated cells, reduced or no phosphorylation on these residues was observed in the CCD- or LB-treated cells (Figs. 2a, b), indicating that disruption of the actin cytoskeleton in G1-phase inhibits integrin-mediated signaling events. Because integrin-mediated cell spreading and organization of the actin cytoskeleton support growth factor signaling, we next investigated whether disruption of actin stress fibers in G1-phase cells also impedes growth factor-induced MAPK phosphorylation. MAPK phosphorylation in untreated cells was detected throughout G1, but was considerably reduced upon treatment with CCD or LB (Fig. 2b), confirming that growth factor signaling in G1 depends on intact actin filaments. Taken together, these results indicate that disruption of the actin cytoskeleton and cell spreading in G1-phase cells attenuates both integrin signaling and growth factor signaling.

### G1/S-phase progression in cycling N2A and CHO cells is not inhibited by disruption of the actin cytoskeleton and cell spreading

**Fig. 3:**
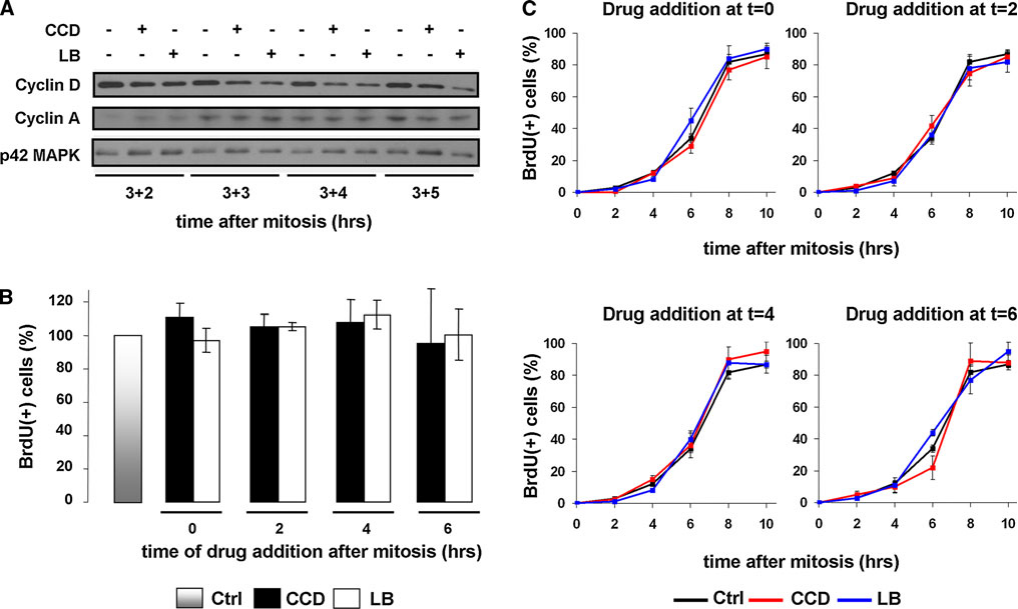
Disruption of actin stress fibers in G1-phase does not inhibit S-phase entry in continuously cycling N2A cells. **a** N2A cells were synchronized by mitotic shake-off and 3 h later treated as described. Cells were lysed 2, 3, 4, and 5 h thereafter and expression of cyclin D and cyclin A was investigated by Western blotting (p42 MAPK = loading control). **b** N2A cells were synchronized by shake-off, released in fresh medium containing 10 μM BrdU, and CCD or LB were added at the indicated time-points. After 14 h, cells were fixed and BrdU incorporation was determined with an ELISA. Incorporation in untreated cells was set to 100 %. The *graph* represents the averages ± SEM from three independent experiments. **c** Mitotic N2A cells were released on coverslips in fresh medium containing 10 μM BrdU, and CCD or LB were added at 0, 2, 4, or 6 h after mitosis. Incorporation of BrdU at the indicated time-points was determined by immunofluorescence

We next examined whether cell cycle progression into S-phase was affected by the disruption of actin filaments. First, we treated cells with the drugs starting at 3 h after mitosis until well into S-phase (8 h post-mitosis), and then analyzed the expression of cyclin D by Western blotting. Consistent with previous observations, a progressive decline in cyclin D levels was observed in CCD- or LB-treated cells, but expression was not abolished completely (Fig. 3a). Moreover, cytoskeletal disruption did also not impair the induction of cyclin A expression (Fig. 3a). We then analyzed whether these cells progressed through S-phase by releasing synchronized cells in medium containing the thymidine analogue BrdU, and measuring BrdU incorporation 14 h after mitosis using an ELISA. To assess whether there is a specific time-frame in G1 that requires cytoskeletal integrity, CCD or LB were added at 0, 2, 4, or 6 h after synchronization. Interestingly, drug addition at neither time-point decreased BrdU incorporation as compared to untreated cells, indicating that also in the presence of the drugs, the entire cell population had entered and completed S-phase (Fig. 3b). In contrast, disruption of the actin cytoskeleton did prevent S-phase entry in quiescent cells that were serum-stimulated to re-enter the cell cycle (Suppl. Fig. 1), as has been documented extensively in a variety of cell types [7, 9, 10, 12, 13, 15]. We then investigated whether the drugs caused a delay or acceleration in the rate of progression from mitosis to S-phase, by analyzing BrdU incorporation at several time-points for up to 10 h after mitosis. BrdU incorporation was identical in all conditions, suggesting that disruption of the cytoskeleton does not affect the rate of G1-phase progression (Fig. 3c).

To exclude that the observed phenomenon is restricted to N2A cells, we next analyzed the same events in CHO cells isolated by mitotic shake-off. Similar to in N2A cells, incubation of spread post-mitotic CHO cells with CCD or LB induced cell rounding and disruption of stress fibers and FAs (data not shown). Correspondingly, FAK autophosphorylation and MAPK phosphorylation were also reduced in CHO cells (Suppl. Fig. 2A). However, as in N2A, progression to S-phase was not prevented, as indicated by expression levels of cyclin A and BrdU incorporation, and no actin cytoskeleton-dependent time-window for G1/S-phase progression was detected (Suppl. Fig. 2B). Together, these results suggest that disruption of the actin cytoskeleton during G1-phase does not inhibit progression from mitosis to S-phase in cycling N2A and CHO cells, despite decreased integrin signaling and growth factor-stimulated MAPK phosphorylation.

### G1/S-phase progression in the absence of cell spreading or stress fibers depends on MAPK signaling

**Fig. 4:**
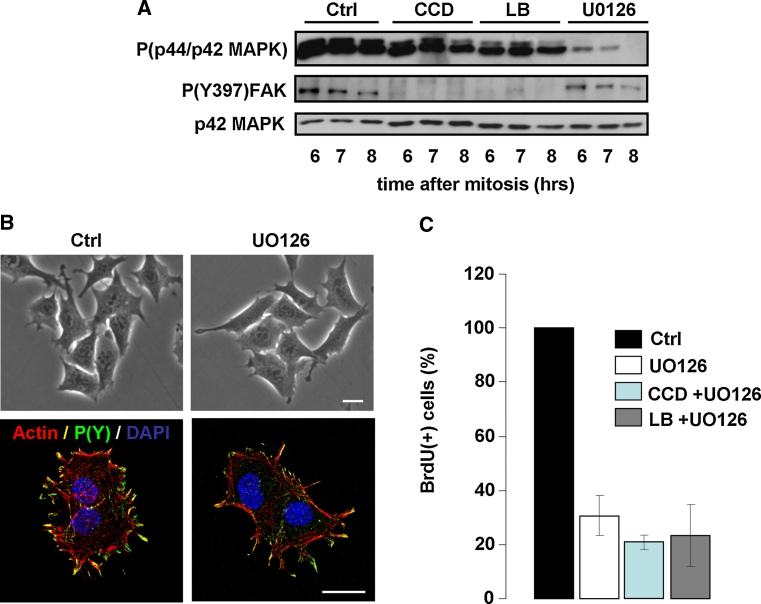
Progression through G1-phase in the absence of cell spreading and stress fibers depends on MAPK signaling. **a** N2A cells were synchronized by shake-off and released in fresh medium supplemented with 500 ng/ml CCD, 100 ng/ml LB, or 20 μM UO126. Cells were lysed at the indicated time-points after synchronization, and phosphorylation of p44/p42 MAPK and (Y397)FAK were investigated by Western blotting (p42 MAPK = loading control). **b** *Top* phase-contrast images of N2A cells that were incubated for up to 5 h after mitosis in the absence (*left*) or the presence of 20 μM UO126 (*right*). *Bar* 10 μm. Bottom; Confocal images showing F-actin (*red*), FAs (visualized with an antibody against phosphotyrosines, *green*) and nuclei (*blue*) in N2A cells that were incubated for up to 5 h after mitosis in the absence (*left*) or the presence of 20 μM UO126 (*right*). *Bar* 10 μm. **c** Mitotic N2A cells were released on coverslips in fresh medium containing either 20 μM UO126 alone, 20 μM UO126 and CCD (500 ng/ml), or 20 μM UO126 and LB (100 ng/ml). BrdU incorporation was then determined 14 h later by immunofluorescence. The *graph* represents the averages from ~300 cells ± SEM from three independent experiments

The previous sections have shown that in the absence of cell spreading, actin stress fibers or detectable FAs, cycling N2A and CHO cells can progress from mitosis to S-phase, despite strongly reduced MAPK activity. Because normal progression from mitosis to S-phase requires sustained MAPK activity throughout G1-phase (Fig. 1d) [33], we next explored the cross-talk between MAPK signaling and cytoskeletal organization, and the requirement for MAPK activity in cytoskeleton-disrupted cells. We therefore synchronized N2A cells by mitotic shake-off and released them in medium containing 500 ng/ml CCD, 100 ng/ml LB, or 20 μM UO126. Western blotting for phosphorylated p44/p42 MAPK levels revealed that UO126 inhibited MAPK activity much more rigorously than CCD and LB, as phospho-MAPK was virtually absent after prolonged incubation with UO126, even on overexposed blots (Fig. 4a). In contrast, P(Y397)FAK was strongly inhibited by CCD or LB but not by UO126, indicating that integrin-dependent events do not require MAPK activity (Fig. 4a) Consistent with this notion, cell spreading, stress fiber formation, or FA assembly (visualized using an antibody against phosphotyrosines) were not affected by treatment with UO126 (Fig. 4b). We then incubated cells in the presence of CCD or LB together with UO126, and determined whether these cells progressed into S-phase by measuring BrdU incorporation as described above. Intriguingly, the addition of UO126 strongly prevented BrdU incorporation, indicating that cell cycle progression in cytoskeleton-disrupted cells critically depends on MAPK activity (Fig. 4c). Taken together, these data indicate that MAPK activity is not required for cell spreading, cytoskeletal organization, and integrin signaling. However, MAPK is crucial for cell cycle progression, both in untreated and cytoskeleton-disrupted cells.

### Cycling N2A cells progress through G2- and M-phase in the absence of cell spreading, actin stress fibers, or focal adhesions

**Fig. 5:**
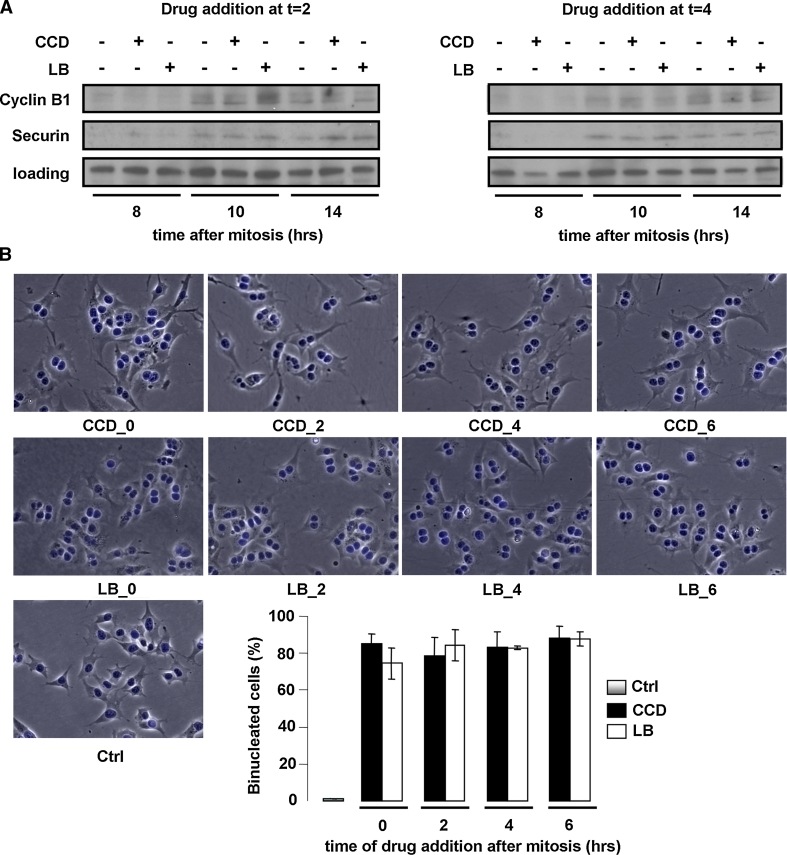
Cycling N2A cells progress through G2- and M-phase in the absence of cell spreading or cytoskeletal integrity. **a** N2A cells were collected by shake-off and treated with CCD or LB at 2 or 4 h after synchronization. Cells were lysed at 8, 10, or 14 h after synchronization, and expression of cyclin B1 and securin were detected by Western blotting. **b** N2A cells were collected by shake-off and treated with CCD or LB at the indicated time-points after synchronization. The drugs were washed away after 20 h, after which the cells were released in fresh medium for 1 h. They were then fixed, stained with DAPI, and the percentage of mono- and bi-nucleated cells was determined from ~300 cells per experiment. The *graph* represents the averages ± SEM from three independent experiments

**Fig. 6:**
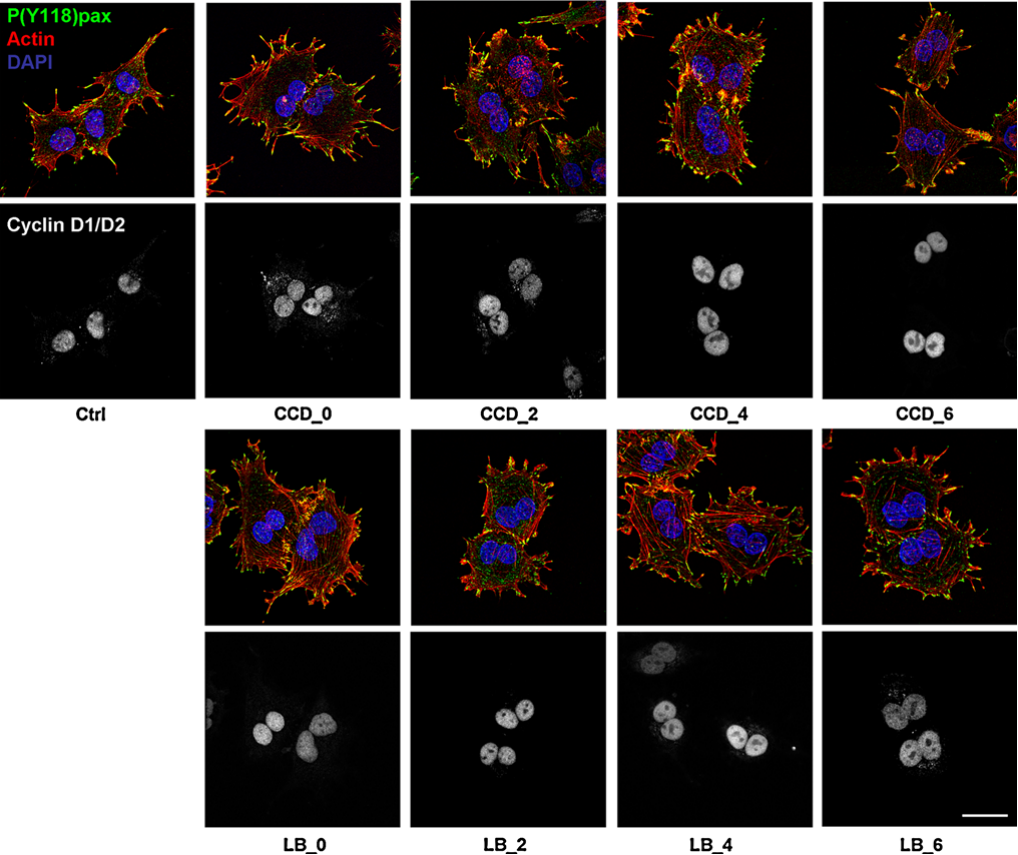
Cell spreading and actin stress fibers are dispensable for nuclear translocation of cyclin D1/D2 in cycling N2A cells. N2A cells were synchronized by shake-off and treated with CCD or LB at the indicated time-points after synchronization. After 20 h, the drugs were washed away and the cells were fixed 1 h later, after which they were processed for microscopy using phalloidin-TRITC and DAPI, as well as antibodies against cyclin D1/D2 and P(Y118)paxillin. Pictures were obtained on a confocal microscope. *Top* nuclei (*blue*), F-actin (*red*), P(Y118)paxillin (*green*). *Bottom* cyclin D1/D2. *Bar* 10 μm

**Fig. 7:**
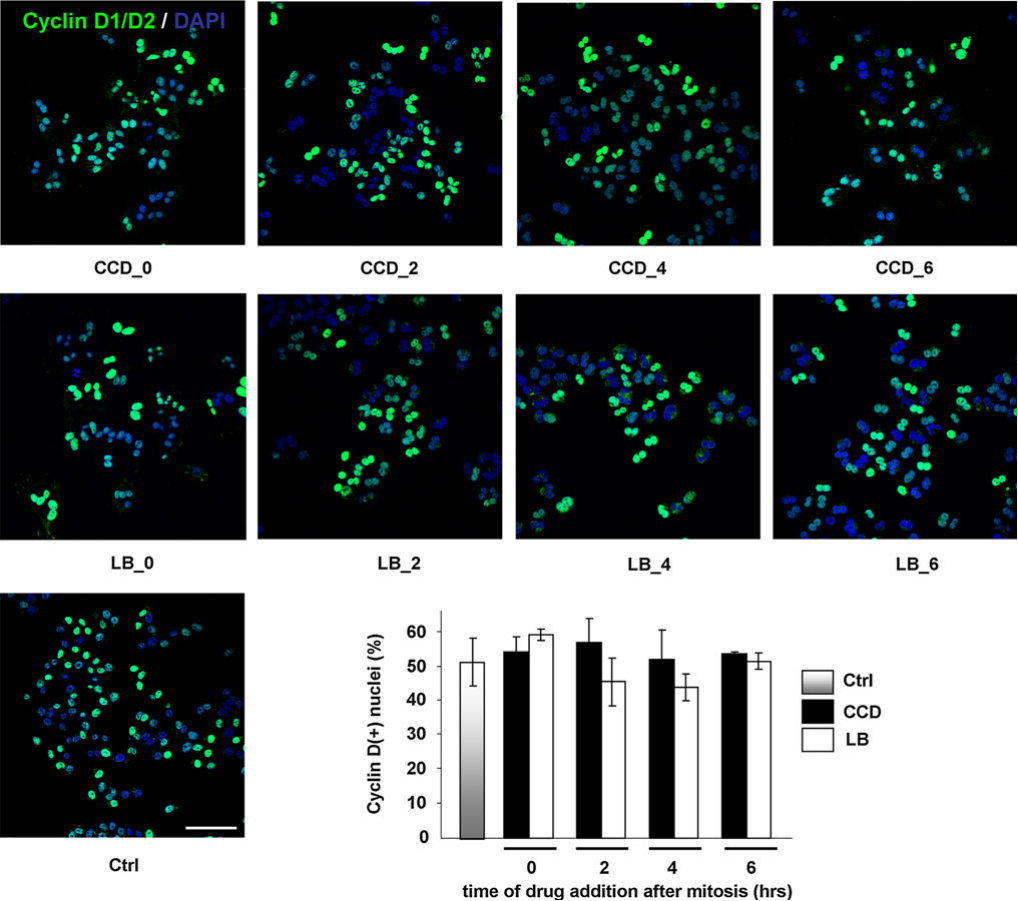
Disruption of cytoskeletal integrity and cell spreading does not prevent entry into a new cell cycle. N2A cells were collected by shake-off and treated with CCD or LB at the indicated time-points after synchronization. After 20 h, the cells were fixed and cyclin D1/D2 (*green*) and the nuclei (*blue*) were visualized with confocal microscopy. The percentage of cyclin D(+) cells was quantified using ImageJ from confocal images acquired with the same settings. For each condition, ~250 cells were analyzed and the *graph* represents the averages ± SEM from three independent experiments. *Bar* 50 μm

We next investigated whether cell cycle progression proceeded after S-phase through G2 and the next M-phase. For this purpose, synchronized cells were treated with the actin inhibitors at several time-points in G1 as described above, and the expression of the G2/M markers cyclin B1, which together with cdk1 promotes entry into mitosis, and securin, an essential modulator of metaphase-anaphase transition, was determined by Western blotting. Intriguingly, both cyclin B1 and securin were detectable from 10 h after mitosis in all conditions, suggesting that cells with disrupted cytoskeletons progress normally though G2-phase and complete their whole cycle (Fig. 5a and data not shown). We then incubated synchronized cells for the length of more than an entire cell cycle with the actin inhibitors. Suppression of actin polymerization during mitosis leads to cleavage failure, creating bi-nucleated cells. We therefore used bi-nucleation as a parameter for cell cycle completion in the presence of CCD or LB. After 20 h of incubation, cells were still rounded, indicating that the inhibitors were still functional (data not shown). The drugs were then washed out, and the cells were allowed to recover for 1 h in fresh medium, in which cell spreading was resumed (Fig. 5b). Cell spreading was accompanied by de novo re-organization of the actin cytoskeleton into stress fibers, as well as FA assembly and integrin signaling, as judged by staining for phosphorylated (Y118)paxillin (Fig. 6). In line with the expression of cyclin B1 and securin, the vast majority of the CCD- and LB-treated N2A cells was bi-nucleated, confirming that they had indeed progressed through G2- and M-phase (Fig. 5b). Moreover, immunofluorescence analysis revealed that all bi-nucleated cells had incorporated BrdU (Suppl. Fig. 3). This is in line with the results obtained with the ELISAs, and reaffirms that the bi-nucleated cells have gone through an entire cycle. Intriguingly, nuclear localization of cyclin D was detected in bi-nucleated cells (Fig. 6). Nuclear import of cyclin D occurs in growth-committed cells in G1-phase and is necessary for passage of the restriction point, whereas its subsequent export and degradation in the cytoplasm is required during S-phase [38]. During the ongoing cell cycle, nuclear cyclin D is detectable starting 2 h after mitosis until the onset of S-phase (~6 h after mitosis), whereafter nuclear cyclin D levels decrease (Suppl. Fig. 4). Quantification of cyclin D-positive nuclei revealed that cyclin D accumulation in the nucleus of CCD- or LB-treated cells was similar to that in untreated cells (Fig. 7), suggesting that these cells not only completed an entire cycle, but even committed to a new one in the presence of the drugs. However, during prolonged treatment with the inhibitors over several days, the multi-nucleated population underwent massive apoptosis, possibly due to genomic instability (data not shown). Summarizing, the data presented here show that N2A and CHO cells can progress through the continuous cell cycle in the absence of extensive cell spreading, stress fibers, or FAs.

### Cycling GEβ1 cells do not require cell spreading, actin stress fibers, or focal adhesions

**Fig. 8:**
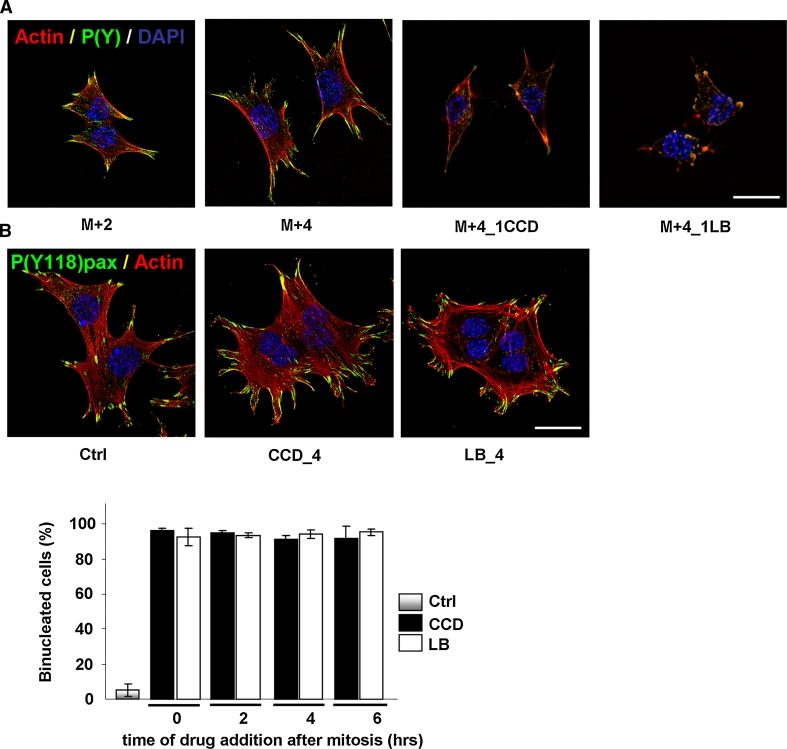
Cell cycle progression in GEβ1 cells does not require cell spreading, actin stress fibers, or FAs. **a** GEβ1 cells were synchronized by mitotic shake-off, released in fresh medium on FN-coated coverslips, and then fixed in G1-phase at the indicated time-points. Alternatively, they were treated with CCD (250 ng/ml) or LB (100 ng/ml) 3 h after mitosis, and fixed 1 h later. The nuclei (*blue*), F-actin (*red*), and FAs (stained with an antibody against phosphotyrosines; *green*) were visualized with confocal microscopy. *Bar* 10 μm. **b** (*top*) GEβ1 cells were synchronized by shake-off and treated with CCD or LB at the indicated time-points after synchronization. After 20 h, the drugs were washed away and the cells were released in fresh medium. They were fixed 1 h later, and nuclei (*blue*), F-actin (*red*), and P(Y118)paxillin (*green*) were visualized with confocal microscopy. *Bar* 10 μm. *Bottom* GEβ1 cells were treated as in **b**, fixed, and stained with DAPI. The percentage of mono- and bi-nucleated cells was determined from ~300 cells per experiment. The *graph* represents the averages ± SEM from three independent experiments

In the previous sections, we have shown that FN-supported cell cycle progression in cycling N2A and CHO cells does not depend on an intact cytoskeleton or cell spreading, which is in apparent contrast to studies using non-transformed capillary endothelial cells and fibroblasts, in which cell cycle re-entry from quiescence is inhibited upon disruption of the actin cytoskeleton [7–14]. The tensional requirements for cell cycle progression may differ between non-transformed and transformed cells. We therefore analyzed FN-supported cell cycle progression under the same conditions in GEβ1 cells, which predominantly express FN-binding integrin α5β1, and do not display typical hallmarks of oncogenic transformation (they do not grow without growth factors or in soft agar, and do not form tumors in mice unless transformed with oncogenic Ras) [35, 39, 40]. Mitotic GEβ1 cells were isolated by shake-off and released on FN, after which they were treated with CCD (250 ng/ml) or LB (100 ng/ml) at appropriate time-points in G1. Cytoskeletal organization, FA assembly, and integrin-induced signaling events were visualized using phalloidin and an antibody against tyrosine-phosphorylated proteins. Post-mitotic GEβ1 cells resumed cell spreading and re-gained stress fibers and FAs within 2 h, and cell spreading increased progressively thereafter (Fig. 8a). Treatment with either drug in G1-phase abolished actin stress fibers, cell spreading, FA assembly, and tyrosine phosphorylations within 1 h (Fig. 8a). We next investigated cell cycle progression in the presence of the inhibitors, by determining the bi-nucleation index as described above. Drugs were washed away 20 h after mitosis, and cells were allowed to recover in fresh medium. A complete recovery of actin stress fibers, cell spreading, and FA assembly was observed 1.5 h after drug wash-out, and the vast majority of the cells appeared to have two nuclei (Fig. 8b). Indeed, bi-nucleation indices clearly show that disruption of the actin cytoskeleton at neither time-point in G1-phase induced a cell cycle arrest (Fig. 8c). Taken together, these data show that similar to in N2A and CHO cells, cell cycle progression in cycling GΕβ1 cells does not depend on actin stress fibers, FAs, or extensive cell spreading.

## Discussion

In this study, we investigated the requirements for FA assembly, cytoskeletal integrity, cell spreading, and MAPK activity in FN-supported progression through the ongoing cell cycle. Mitotic N2A and CHO cells were collected by shake-off and released on FN-coated dishes, after which post-mitotic actin stress fiber formation was abolished with pharmacological agents at progressive time-points during G1-phase. Cell spreading, FA assembly, and integrin signaling were disrupted by this treatment. Furthermore, growth factor-induced p44/p42 MAPK phosphorylation was also considerably decreased, confirming the well-established link between integrin-mediated cytoskeletal organization and growth factor signaling [41–43]. Consequently, we observed a decline in cyclin D levels, in line with a number of reports showing that optimal cyclin D expression is jointly regulated by growth factors and integrin-mediated cell spreading and cytoskeletal organization [5, 12–15, 26–28]. However, the residual cyclin D levels were apparently sufficient to support G1-phase progression into S-phase, since expression of cyclin A was not prevented, and virtually all cells committed to DNA synthesis. It thus seems that for cells that enter G1-phase from mitosis, G1-phase progression does not depend on cytoskeletal tension. In fact, the vast majority of the cells completed the entire cycle in the absence of detectable actin filaments, giving rise to a population of giant bi-nucleated cells due to absence of the actin contractile ring that separates the daughter cells during cytokinesis.

The results presented here complement our previous study, and suggest that cycling cells do not require an intact cytoskeleton or cell spreading, as long as expression of cyclin D is maintained. Furthermore, we now show that nuclear translocation of cyclin D during G1-phase of continuously cycling cells occurs independently of cell spreading/cytoskeletal organization. Thus, cyclin D expression (but not its nuclear translocation) depends on cell spreading and the cytoskeleton, which is in line with observations in cells that were growth factor-stimulated to re-enter G1-phase from G0 [44, 45]. Nuclear translocation of cyclin D is a key event in G1-phase that is required for phosphorylation of the retinoblastoma protein and passage of the restriction point [37]. However, export of cyclin D out of the nucleus and its subsequent cytoplasmic degradation are important for normal passage through S-phase [46, 47]. It is thus paramount that nuclear trafficking of cyclin D is tightly regulated. Indeed, whereas overexpression of cyclin D is observed in many tumor cells, it is by itself not sufficient for oncogenic transformation, and accumulating evidence suggests that transformation is driven by nuclear retention of cyclin D [38]. Strategies that target the nuclear trafficking of D-type cyclins rather than merely their expression may therefore prove to be effective in the treatment of cancer. Because the expression of cyclin D is tightly linked to MAPK activity, and sustained MAPK activity throughout G1-phase until the restriction point is important for integrin-regulated cell cycle progression, an interesting question that arises from our data is whether the observed cell cycle progression in cells with disorganized cytoskeletons has become independent of MAPK. To delineate the cross-talk between MAPK, the cytoskeleton, and cell spreading in cycling cells, we inhibited the MAPK pathway using the inhibitor UO126. UO126 robustly inhibited progression to S-phase, but cell morphology and cytoskeletal organization, as well as FA assembly and integrin-stimulated autophosphorylation of FAK were not affected. Thus, although cell spreading and the cytoskeleton potentiate MAPK activation, the MAPK pathway seems not reciprocally involved in integrin signaling and integrin-mediated cell spreading and cytoskeletal organization. Intriguingly, cell cycle progression in cytoskeleton-disrupted cells was also inhibited by UO126. These data thus identify the MAPK pathway as the mechanism that drives proliferation in the absence of cell spreading and cytoskeletal tension, and suggest that cell cycle progression can be uncoupled from tensional and morphological requirements by MAPK activation. It has been suggested that the tension-requirement for cell cycle progression differs between transformed and non-transformed cells, based on the observation that re-entry into G1-phase from quiescence is prevented by cytoskeletal disruption in non-transformed primary capillary endothelial cells and NIH3T3 fibroblasts overexpressing integrin α5β1 [5]. Indeed, transformation induces cell rounding and a loss of actin stress fibers, and a number of early reports have shown that actin-destabilizing agents do not affect proliferation in cycling transformed cells [48–50]. We therefore aimed to investigate whether integrin α5β1-supported cell cycle progression depends on cytoskeletal organization and cell spreading in cycling non-transformed cells. First, we generated NIH3T3 cells that stably overexpress α5β1, however shake-off experiments with this cell line did not yield sufficient numbers of mitotic cells (data not shown). We then chose GEβ1 cells as a model system, because they (1) predominantly express integrin α5β1 whereas the expression of other integrins is negligible, (2) display a well-spread, fibroblast-like shape with extensive actin stress fibers and FAs, (3) do not grow without growth factors or in soft agar, and (4) are importantly not tumorigenic in mice unless transformed with oncogenic Ras [40]. We thus consider GEβ1 cells to be a non-transformed cell line, at least with respect to growth-control mechanisms and cell cycle requirements. Intriguingly, cell cycle progression in cycling GEβ1 cells was not impaired by disruption of cytoskeletal integrity, cell spreading, and FA assembly in G1-phase, similar to what we have observed in CHO and N2A cells. We therefore conclude that cycling mammalian cells, transformed and non-transformed alike, can proliferate in the absence of cytoskeletal tension or cell spreading, provided that there is MAPK signaling to sustain threshold levels of cyclin D. Nevertheless, it is likely that cell cycle regulation in different cell types involves different regulatory mechanisms. For example, regulation of cell proliferation by cell shape and cytoskeletal tension has been well documented for fibroblasts and endothelial cells, which adopt a more “tensile” morphology than epithelial cells. Indeed, E-cadherin signals rather than cell-matrix interactions can drive cyclin D expression in epithelial cells [51]. Furthermore, whereas stress fibers are not commonly observed in cells in vivo, specialized cell types as wound fibroblasts and myofibroblasts develop them under conditions of great mechanical stress which require both proliferation and contraction, such as wound healing [52]. Probably, these cell types retain the tensional requirement for cell cycle entry from quiescence in vitro.

## Electronic supplementary material

Below is the link to the electronic supplementary material.

Suppl Fig 1 Disruption of actin stress fibers inhibits S-phase entry in serum-stimulated quiescent cells but not in cycling cells. N2A cells were synchronized by mitotic shake-off and mitotic cells were released in medium with serum, CCD (500 ng/ml), and 10 μM of the thymidine analogue EdU (a). In parallel, N2A cells that had been serum-starved for 36 hrs were trypsinized and released in medium with serum, CCD (500 ng/ml), and 10 μM of the thymidine analogue EdU (b). Incorporation of EdU (green) was visualized 10 hrs later by confocal microscopy, using the Click-iT EdU imaging kit. Nuclei were counter-stained with DAPI (blue). The arrow indicates a cell that has incorporated EdU (TIFF 1400 kb)

Suppl Fig 2 Disruption of actin stress fibers in G1-phase does not inhibit S-phase entry in cycling CHO cells. (a) CHO cells were synchronized by mitotic shake-off, released in fresh medium, and treated 3 hrs after synchronization with 500 ng/ml CCD or 100 ng/ml LB. Cells were lysed 2, 3, 4 and 5 hrs thereafter and autophosphorylation of (Y397)FAK, as well as phosphorylation of p44/p42 MAPK were investigated by Western blotting (p42MAPK=loading control). (b) (Top) CHO cells were synchronized by mitotic shake-off and 3 hrs later treated as descibed. Cells were lysed 5, 6, and 7 hrs thereafter, and expression of cyclin A was investigated by Western blotting (p42 MAPK=loading control). (Bottom) Synchronized CHO cells were released in medium containing 10 μM BrdU, and CCD or LB were added at the indicated time-points. Cells were fixed 14 hrs thereafter and BrdU incorporation was determined using an ELISA. Incorporation in untreated cells was set to 100%. The graph represents the averages ± s.e.m. from 3 independent experiments (TIFF 979 kb)

Suppl Fig 3 Binucleated cells have incorporated BrdU. N2A cells were synchonized by shake-off, released in medium containing 10 μM BrdU, and treated with CCD or LB at the indicated time-points after synchronization. After 20 hrs, they were fixed and processed for microscopy using an antibody against BrdU, whereas nuclei were counter-stained with DAPI. Images were acquired on a confocal microscope. (Top) BrdU, (bottom) DAPI (TIFF 2285 kb)

Suppl Fig 4 Nuclear translocation of cyclin D in early G1-phase. N2A cells were synchronized by mitotic shake-off and released on coverslips as described in materials and methods. At the indicated time-points thereafter, cells were fixed, processed for microscopy using an antibody against cyclin D1/D2 and DAPI, and images were captured on a confocal microscope (TIFF 1152 kb)

## Abbreviations

CCD: Cytochalasin D
ECM: Extracellular matrix
F-actin: Filamentous actin
FA: Focal adhesion
FAK: Focal adhesion kinase
FN: Fibronectin
LB: Latrunculin B
MAPK: Mitogen-activated protein kinase
