# Supplementary figures and images for: MAPK uncouples cell cycle progression from cell spreading and cytoskeletal organization in cycling cells
Source: Cell Mol Life Sci. 2012 Aug 25;70(2):293–307. doi: 10.1007/s00018-012-1130-2 (PMC3535415; doi:10.1007/s00018-012-1130-2)

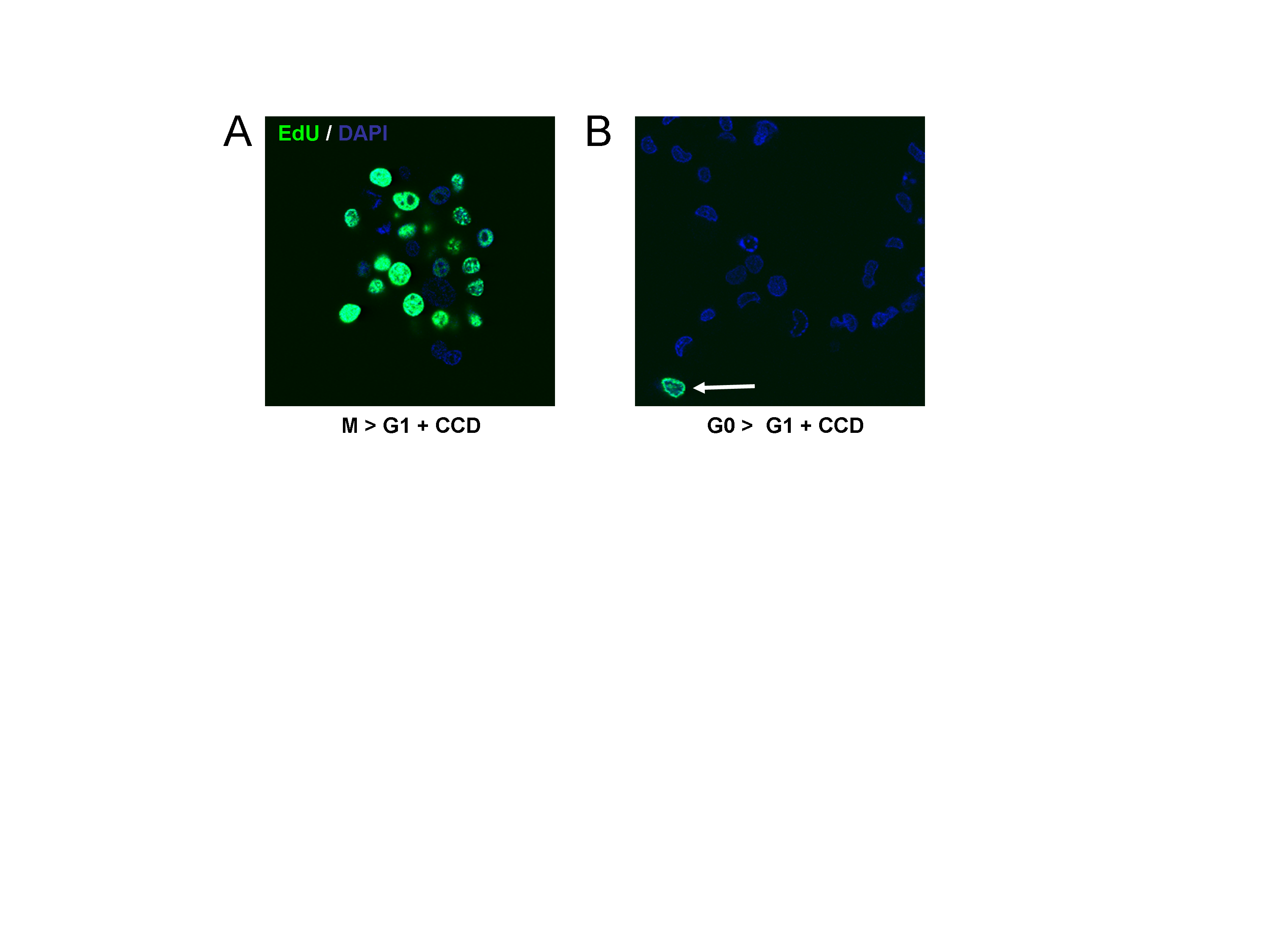

Supplement: Supplementary file 1 — Suppl Fig 1 Disruption of actin stress fibers inhibits S-phase entry in serum-stimulated quiescent cells but not in cycling cells. N2A cells were synchronized by mitotic shake-off and mitotic cells were released in medium with serum, CCD (500 ng/ml), and 10 μM of the thymidine analogue EdU (a). In parallel, N2A cells that had been serum-starved for 36 hrs were trypsinized and released in medium with serum, CCD (500 ng/ml), and 10 μM of the thymidine analogue EdU (b). Incorporation of EdU (green) was visualized 10 hrs later by confocal microscopy, using the Click-iT EdU imaging kit. Nuclei were counter-stained with DAPI (blue). The arrow indicates a cell that has incorporated EdU (TIFF 1400 kb) [file 18_2012_1130_MOESM1_ESM.tif]

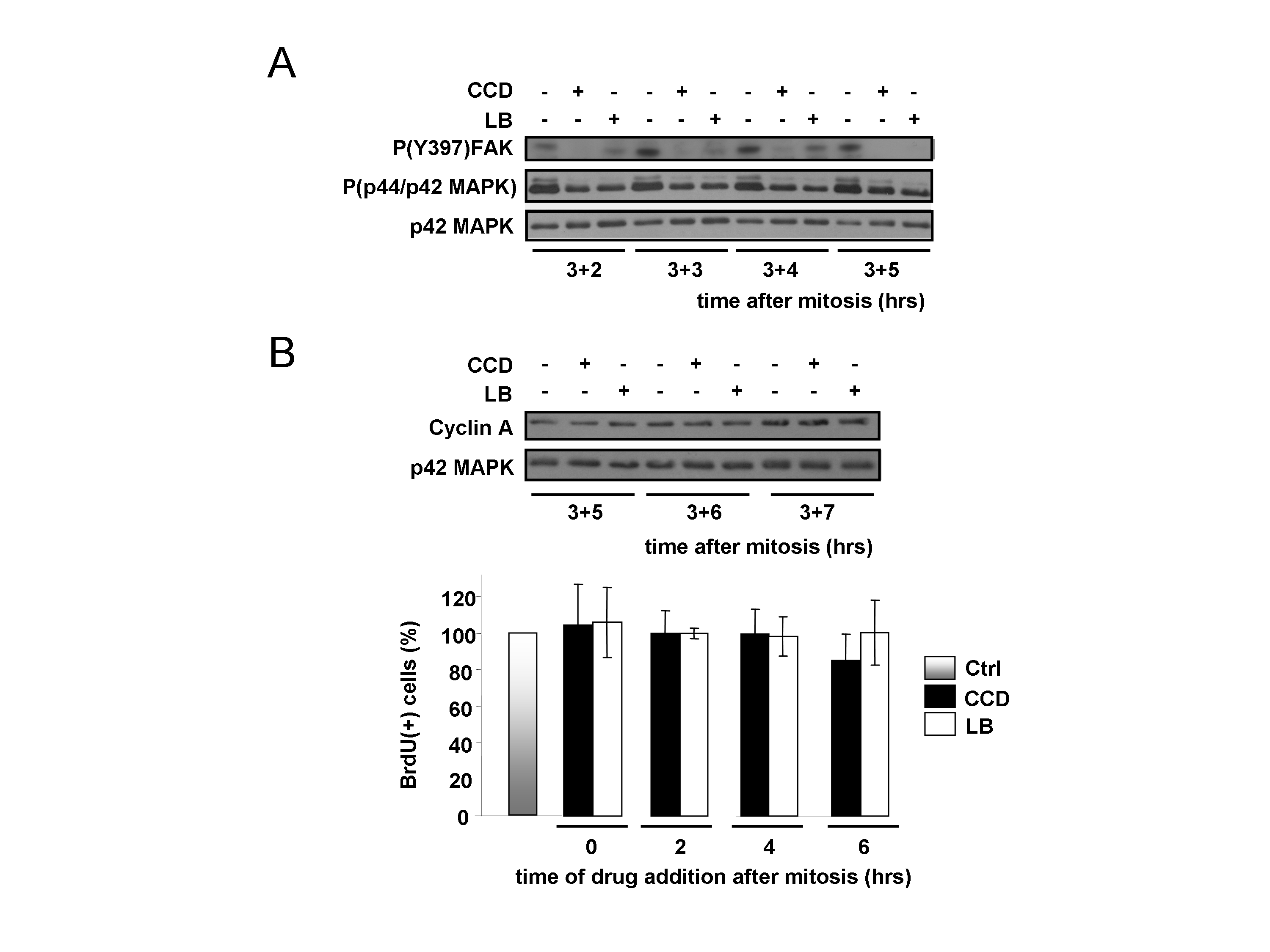

Supplement: Supplementary file 2 — Suppl Fig 2 Disruption of actin stress fibers in G1-phase does not inhibit S-phase entry in cycling CHO cells. (a) CHO cells were synchronized by mitotic shake-off, released in fresh medium, and treated 3 hrs after synchronization with 500 ng/ml CCD or 100 ng/ml LB. Cells were lysed 2, 3, 4 and 5 hrs thereafter and autophosphorylation of (Y397)FAK, as well as phosphorylation of p44/p42 MAPK were investigated by Western blotting (p42MAPK=loading control). (b) (Top) CHO cells were synchronized by mitotic shake-off and 3 hrs later treated as descibed. Cells were lysed 5, 6, and 7 hrs thereafter, and expression of cyclin A was investigated by Western blotting (p42 MAPK=loading control). (Bottom) Synchronized CHO cells were released in medium containing 10 μM BrdU, and CCD or LB were added at the indicated time-points. Cells were fixed 14 hrs thereafter and BrdU incorporation was determined using an ELISA. Incorporation in untreated cells was set to 100%. The graph represents the averages ± s.e.m. from 3 independent experiments (TIFF 979 kb) [file 18_2012_1130_MOESM2_ESM.tif]

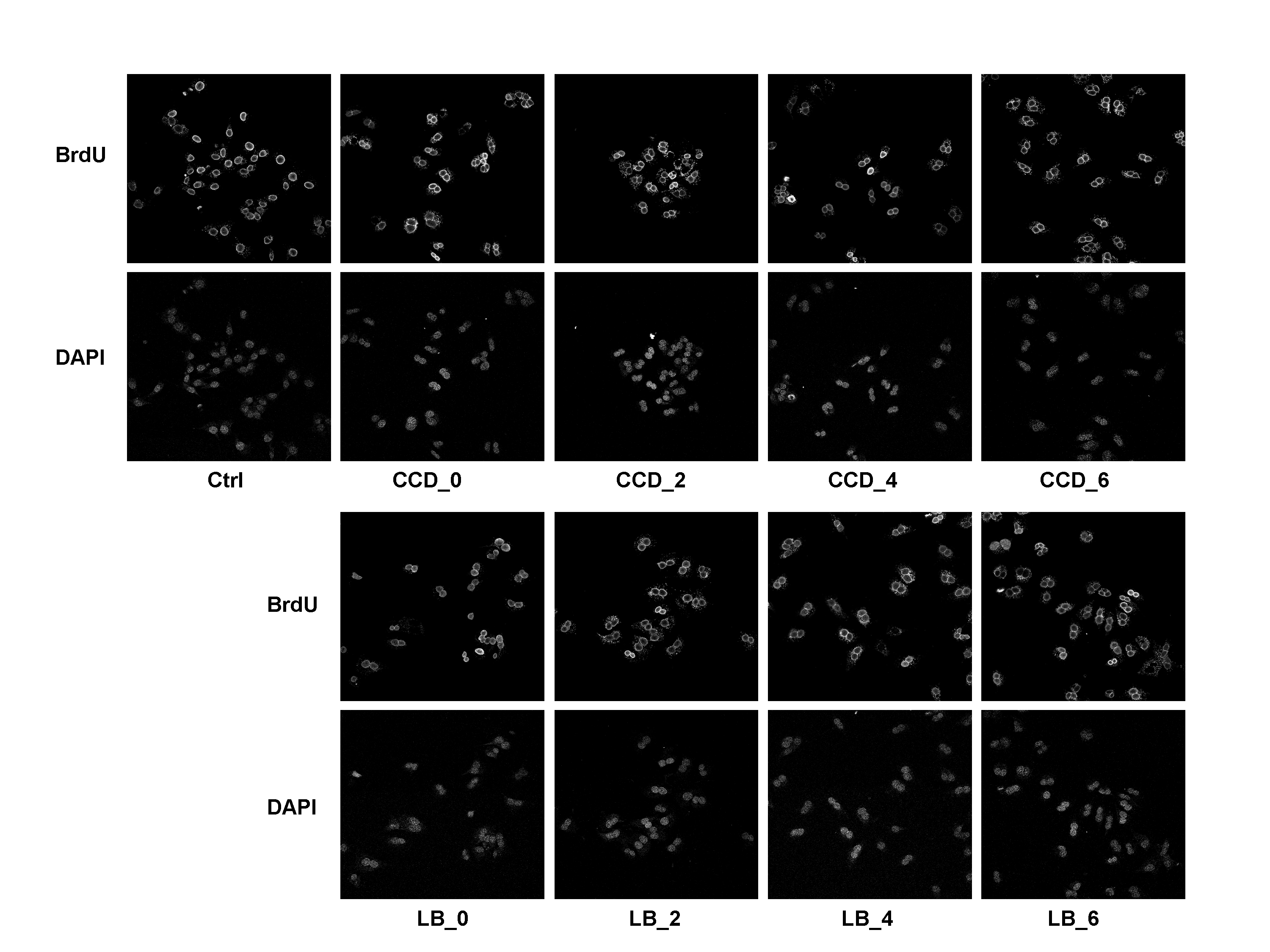

Supplement: Supplementary file 3 — Suppl Fig 3 Binucleated cells have incorporated BrdU. N2A cells were synchonized by shake-off, released in medium containing 10 μM BrdU, and treated with CCD or LB at the indicated time-points after synchronization. After 20 hrs, they were fixed and processed for microscopy using an antibody against BrdU, whereas nuclei were counter-stained with DAPI. Images were acquired on a confocal microscope. (Top) BrdU, (bottom) DAPI (TIFF 2285 kb) [file 18_2012_1130_MOESM3_ESM.tif]

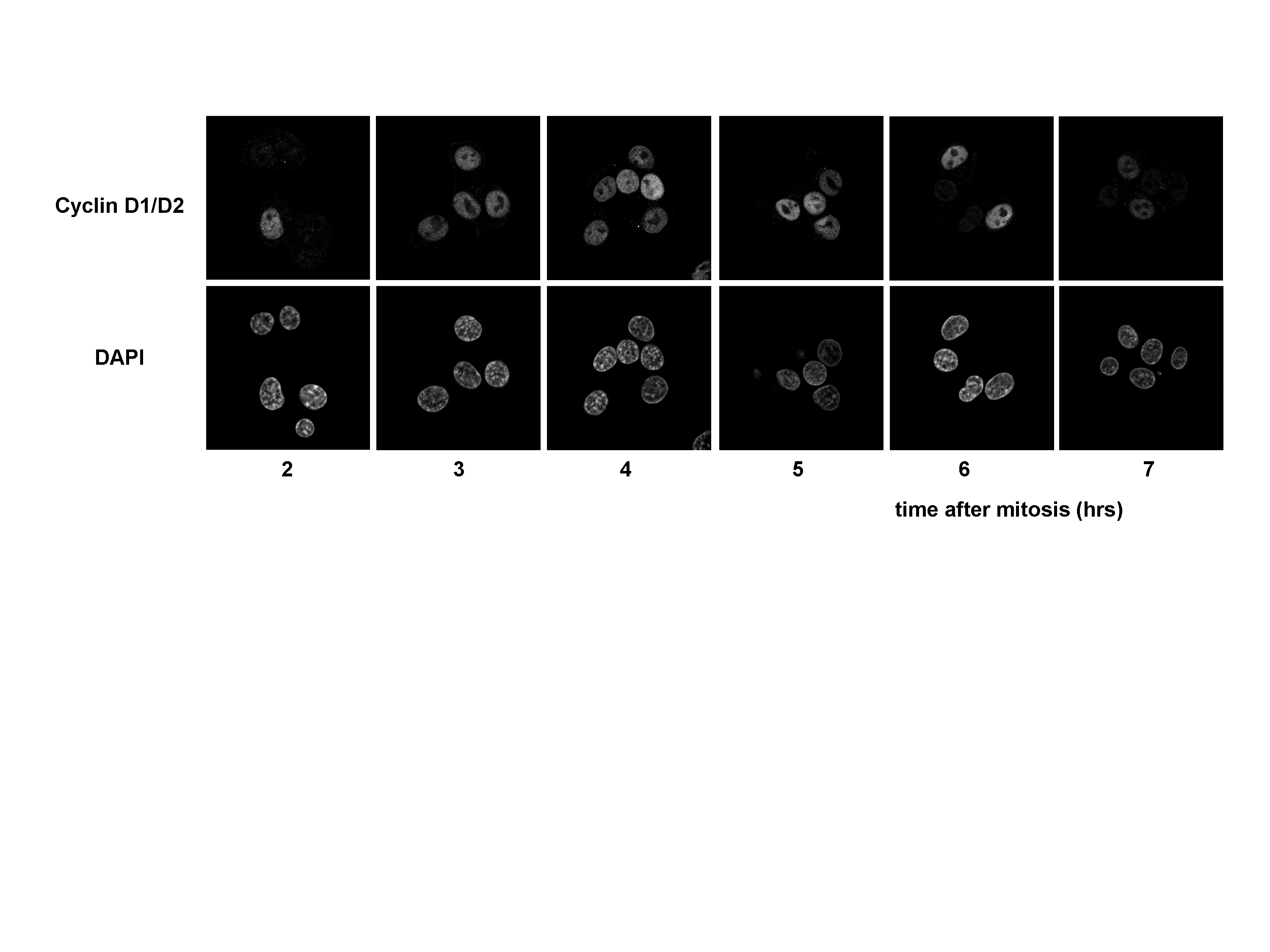

Supplement: Supplementary file 4 — Suppl Fig 4 Nuclear translocation of cyclin D in early G1-phase. N2A cells were synchronized by mitotic shake-off and released on coverslips as described in materials and methods. At the indicated time-points thereafter, cells were fixed, processed for microscopy using an antibody against cyclin D1/D2 and DAPI, and images were captured on a confocal microscope (TIFF 1152 kb) [file 18_2012_1130_MOESM4_ESM.tif]
